# The research progress on effective connectivity in adolescent depression based on resting-state fMRI

**DOI:** 10.3389/fneur.2025.1498049

**Published:** 2025-02-10

**Authors:** Xuan Deng, Jiajing Cui, Jinyuan Zhao, Jinji Bai, Junfeng Li, Kefeng Li

**Affiliations:** ^1^Department of Radiology, Affiliated Heping Hospital, Changzhi Medical College, Changzhi, China; ^2^Artificial Intelligence Drug Discovery Center, Faculty of Applied Sciences, Macau Polytechnic University, Macau, China

**Keywords:** resting-state fMRI, effective connectivity, brain functional networks, depression, adolescent

## Abstract

**Introduction:**

The brain’s spontaneous neural activity can be recorded during rest using resting state functional magnetic resonance imaging (rs-fMRI), and intricate brain functional networks and interaction patterns can be discovered through correlation analysis. As a crucial component of rs-fMRI analysis, effective connectivity analysis (EC) may provide a detailed description of the causal relationship and information flow between different brain areas. It has been very helpful in identifying anomalies in the brain activity of depressed teenagers.

**Methods:**

This study explored connectivity abnormalities in brain networks and their impact on clinical symptoms in patients with depression through resting state functional magnetic resonance imaging (rs-fMRI) and effective connectivity (EC) analysis. We first introduce some common EC analysis methods, discuss their application background and specific characteristics.

**Results:**

EC analysis reveals information flow problems between different brain regions, such as the default mode network, the central executive network, and the salience network, which are closely related to symptoms of depression, such as low mood and cognitive impairment. This review discusses the limitations of existing studies while summarizing the current applications of EC analysis methods. Most of the early studies focused on the static connection mode, ignoring the causal relationship between brain regions. However, effective connection can reflect the upper and lower relationship of brain region interaction, and provide help for us to explore the mechanism of neurological diseases. Existing studies focus on the analysis of a single brain network, but rarely explore the interaction between multiple key networks.

**Discussion:**

To do so, we can address these issues by integrating multiple technologies. The discussion of these issues is reflected in the text. Through reviewing various methods and applications of EC analysis, this paper aims to explore the abnormal connectivity patterns of brain networks in patients with depression, and further analyze the relationship between these abnormalities and clinical symptoms, so as to provide more accurate theoretical support for early diagnosis and personalized treatment of depression.

## 1 Introduction

### 1.1 The epidemiology and social impacts of depression

**Figure 1:**
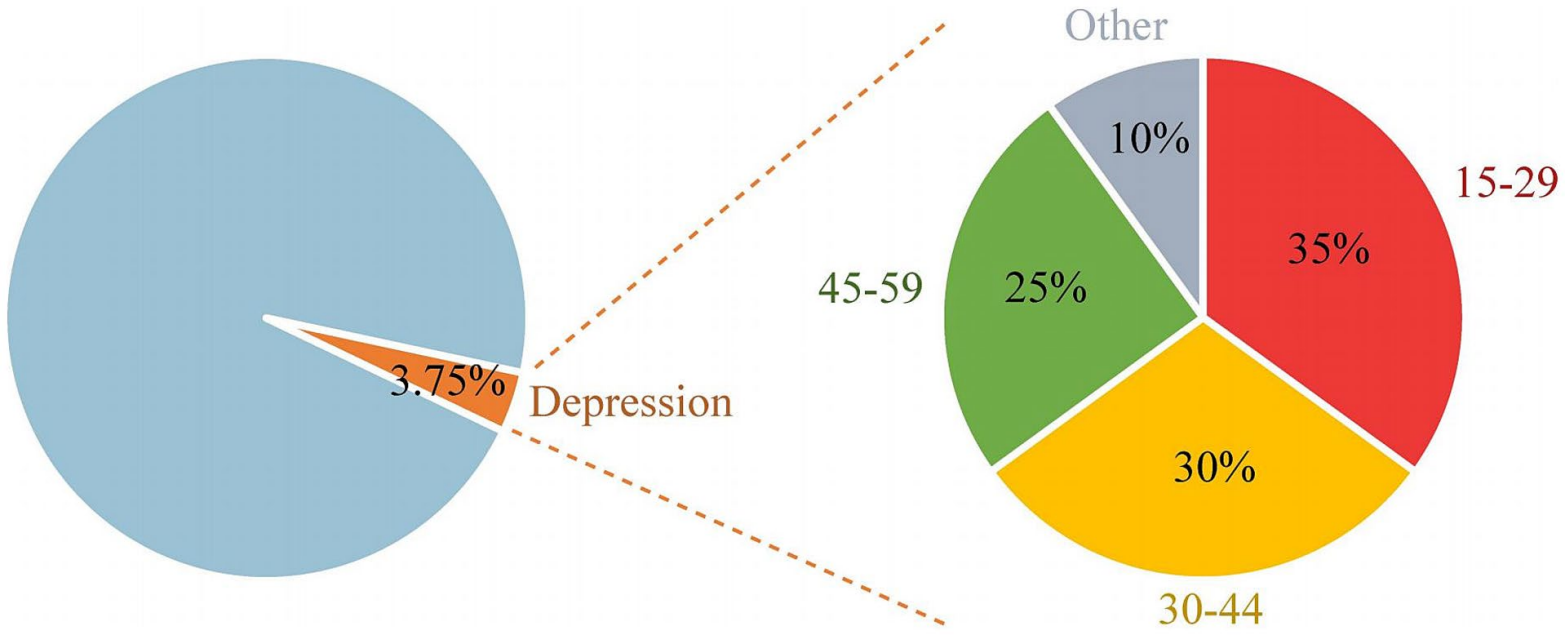
The proportion of global depression cases to the total number of people in 2019 and the proportion of depressive patients by age group.

Over the past few decades, there has been a sharp rise in the number of new instances of depression, which has serious consequences for public health worldwide (1). According to recent statistics reports, depression is more common in women than in men worldwide and is rising (2). The disparity in prevalence between men and women is influenced by a variety of factors, including societal, psychological, and biological aspects. Because adolescent females’ sex hormones alter more dramatically, their prevalence is higher (3). Approximately 290 million individuals worldwide suffer from depression as of 2019, making up 3.75% of the world’s population. The incidence is 30–35% higher in those between the ages of 15 and 29 (4, 5). Depression progressively became one of the main mental illnesses impacting the health of teenagers between 1990 and 2019 (6). Patients suffering from depression may experience severe mental health problems, including sleep disorders, cognitive impairment, executive dysfunction, and an increased risk of suicide (7). Investigating objective indicators of depression is crucial for early diagnosis and therapy because of the shortcomings and subjectivity of the scoring systems currently in use (Figure 1).

### 1.2 The use of resting-state fMRI in depression research

Recently, some studies have proposed that resting-state fMRI has certain application values in the treatment of depression, mainly manifested as follows: First, identifying abnormal signals. Resting-state fMRI can effectively identify abnormalities in brain functional networks. Existing research has confirmed that there are abnormal phenomena in the brain network connections of individuals with depression, mainly appearing in the default mode network (DMN) and the medial prefrontal cortex to the limbic emotional regulation circuit (MRC) (8). These abnormalities affect the subject’s cognitive and emotional regulation. Second, understanding the pathological mechanisms, the onset mechanisms of depression are often related to abnormalities in brain functional networks (9). The promotion and application of resting-state fMRI technology have unveiled the pathological mechanisms of depression for medical professionals (10). Third, powerful in evaluation and prediction, comparing clinical presentations before and after treatment to assess treatment effects. Evaluating the scientific nature and feasibility of medication plans, judging whether brain cognitive and emotional regulation has improved after medication, and also predicting individual medication responses (11). Fourth, comparing patient groups, by comparing different types of onset patients, finding similarities and differences among them, which provides a basis for depression classification and process management (12, 13). Fifth, revealing gender differences, studies show significant differences in brain regions between male and female patients with severe depression (14).

### 1.3 Definition and importance of effective connectivity

Even though functional connectivity (FC) in the brain can now be seen with resting-state functional magnetic resonance imaging (fMRI), it is still challenging to completely comprehend the causal relationships between various brain regions when relying only on correlation analysis of FC. Effective connectivity (EC) becomes very crucial in fMRI research in this situation. As a vital instrument for a thorough investigation of brain function, EC shows how neurons cooperate to provide intricate cognitive and behavioral processes by examining the connections and information flow between various brain regions. In addition, it shows how different parts of the brain interact dynamically, particularly how information moves from the sensory cortex to higher cognitive regions and how strongly. We can better understand the neurobiological mechanisms underpinning early-stage depression by using EC to uncover patterns of information transmission between different parts of the brain in the study of adolescent depression (15). According to recent studies, adolescents with depression have very different EC patterns than adult patients, and these early alterations in functional connectivity could be a predictor of the condition’s long-term course (16). By building and evaluating dynamic models, like Dynamic Causal Modeling (DCM), EC can more accurately represent the driving mechanisms of neural networks than FC, which mainly concentrates on the statistical connections between brain areas. Thus, in addition to conceptually addressing FC’s shortcomings, EC offers a more thorough viewpoint for comprehending the dynamic connections within intricate brain functional networks in real-world applications. Combining FC and EC provides more thorough and extensive theoretical support for the diagnosis and treatment of neurological illnesses by analyzing the brain’s resting state functional organisation and underlying neural mechanisms. In order to achieve early intervention and individualized treatment, it is crucial to conduct a thorough examination of effective connection in young patients with depression. In addition to offering comprehensive data on connection strength, directionality, and time series, EC illustrates the dynamic transfer of information within brain networks by building causal relationship models between different brain areas. For the purpose of comprehending intricate neural processes like perception, memory, and decision-making, this allows researchers to go beyond conventional correlation analyses and investigate how the brain coordinates its activities under various states (such as resting or performing tasks) (17). Alterations in EC often reflect the fundamental pathological mechanisms underlying psychiatric disorders such as depression, schizophrenia, and Alzheimer’s disease (18, 19). Researchers can learn more about how these illnesses impair the regular operation of brain networks by contrasting the EC patterns of patients and healthy people. Moreover, adding structural connectivity (SC) data to EC models can greatly improve the analysis’ accuracy and explanatory capacity (20). Even though resting-state fMRI has shown great promise in the study of depression, the development of efficient connectivity analysis enables a more thorough understanding of the causal relationships within brain networks and their roles in the etiology of depression, especially in patient populations of adolescents. In addition to addressing gaps in the literature, this offers a strong theoretical framework for creating intervention tactics that are more accurate and successful.

### 1.4 Rationale for focusing on three networks and the role of other networks

The salience network (SN), central executive network (CEN), and default mode network (DMN) are the main research objects in this study. Their significant significance in the fundamental mechanisms of depression informed the decision, which was not made by mistake. Abnormal functioning of the DMN, particularly overactivation between the anterior medial prefrontal cortex and the posterior cingulate gyrus, is frequently regarded as a major neural basis for ruminative thinking and negative self-evaluation. The DMN is closely linked to self-reflection and intrinsic emotional regulation. CEN is essential for goal-directed behavior and cognitive regulation. According to the study, patients were more likely to show signs of inattention and trouble completing activities when the connection of the CEN was compromised. The slow or rigid emotional reactions of patients are typically directly linked to the malfunctioning of the SN network, which serves as a “hub” between the DMN and CEN and is in charge of screening and processing important inputs. According to these results, these three networks are essential to the study of depression and are hence the subject of this investigation.

However, we also know that the aforementioned networks are not the only pathogenic processes of depression. However, we also know that the aforementioned networks are not the only pathogenic processes of depression. In certain disease processes, other functional networks—like the limbic system and attention networks—may also be crucial. For instance, the limbic system has a direct role in emotion production and memory integration, whereas attention networks are linked to information filtering and attention management. These networks were excluded from our analysis for a number of reasons. To guarantee the study’s relevance and the depth of its conclusions, this research first sought to concentrate on the networks most strongly linked to the fundamental mechanisms of depression. Second, our chosen dynamic causal modeling approach is best suited for examining the causal relationships between particular networks rather than the intricate relationships among all functional networks. Lastly, in order to guarantee the accuracy of data analysis, we must choose research objects sensibly given the constraints of sample size and research resources. In order to present a more comprehensive map of brain connections and offer more multifaceted theoretical support for the diagnosis and treatment of depression, future research can progressively include these networks in the discussion. These networks were excluded from our analysis for a number of reasons. To guarantee the study’s relevance and the depth of its conclusions, this research first sought to concentrate on the networks most strongly linked to the fundamental mechanisms of depression. Second, our chosen dynamic causal modeling approach is best suited for examining the causal relationships between particular networks rather than the intricate relationships among all functional networks. Lastly, in order to guarantee the accuracy of data analysis, we must choose research objects sensibly given the constraints of sample size and research resources. In order to present a more comprehensive map of brain connections and offer more multifaceted theoretical support for the diagnosis and treatment of depression, future research can progressively include these networks in the discussion.

### 1.5 Method

**Figure 2:**
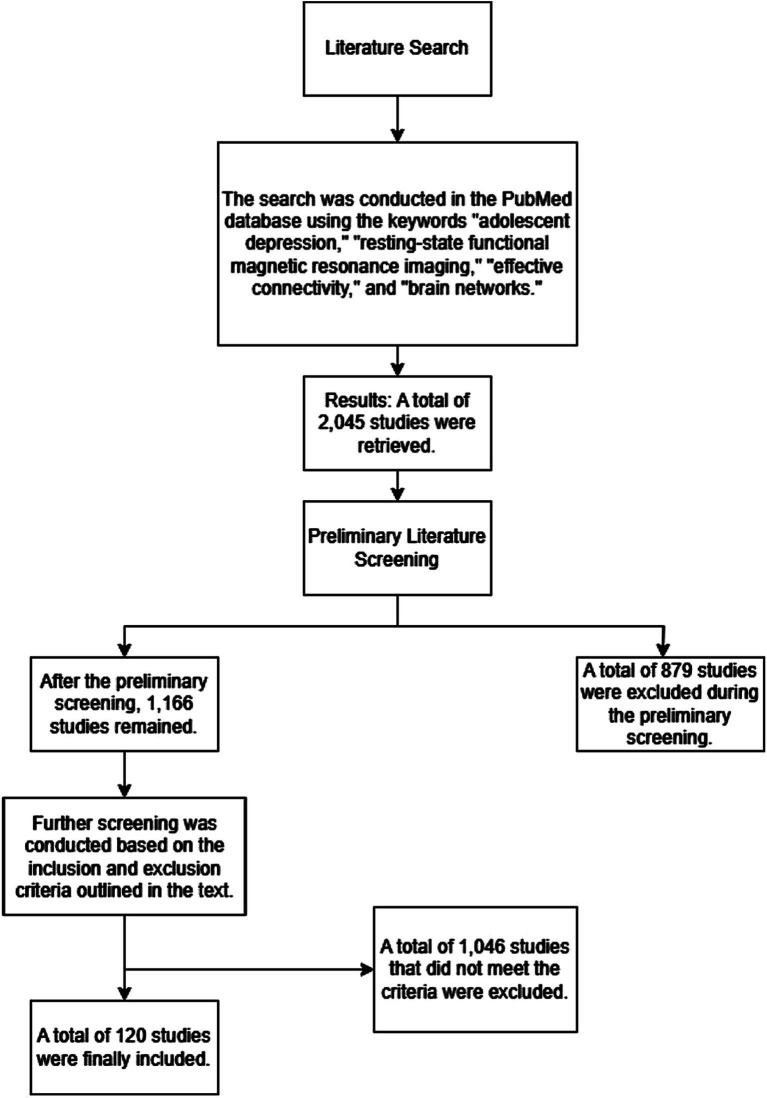
Flowchart of the literature screening process.

This paper reviews the progress of studies on effective connectivity of depression in adolescents based on resting state functional magnetic resonance imaging (rs-fMRI) through systematic review and review analysis. The study participants were primarily adolescents with untreated first episode depression and were compared with healthy controls. 2,045 articles were retrieved from the PubMed database, which included the keywords “adolescent depression,” “resting state magnetic resonance imaging,” “effective connectivity,” and “brain networks.” Then 879 articles were further screened according to inclusion and exclusion criteria (Figure 2).

Qualifications for inclusion:

1. Adolescent brain function during resting state fMRI studies, especially in patients with major depressive disorder (MDD).
2. To determine the causative relationship between brain networks, apply efficient connection analysis techniques (e.g., Granger causal analysis, dynamic causal model, structural equation model, etc.).
3. Examine anomalies in the brain’s functional networks, especially those that are connected to central symptoms like self-talk, emotional control, and cognitive control.
4. The material that has just been published is given priority in order to guarantee cutting-edge technology and timely research.

Termination of the standard:

1. Research that did not employ resting state fMRI or that did not have depression or teenagers as their primary focus were not included. Studies without resting state fMRI were not taken into consideration because it is a crucial non-invasive neuroimaging technique that is frequently used to examine brain network connection.
2. Research that solely examined functional connection without looking at causal connectivity was not accepted. Granger causality and dynamic causal modeling are two examples of causal analysis techniques that were necessary for inclusion since they are crucial for comprehending brain network connections.
3. Due to possible bias and untrustworthy results, research with subpar statistical analysis (such as non-significant results, incorrect methodologies) or inadequate data processing (such as motion artifacts, poor standardization) was disqualified. Excluded studies also lacked a control group or had inadequate sample sizes (less than 10 participants per group).
4. To guarantee the review’s timeliness and accuracy, the more recent or methodologically sound version was given priority when several studies from the same team were available.

## 2 Etiology, characteristics, and neuroimaging studies of adolescent depression

### 2.1 Features and challenges of adolescent depression

Adolescence is a critical period in the development of the human being; during this time, people’s physical, psychological, and social roles will change significantly. Additionally, even small environmental changes at this time may cause significant swings in psychological health, therefore this period of life need special attention. Adolescent depression’s clinical presentations can be somewhat complicated, with a high misdiagnosis rate and a range of symptoms that include loss of interest and self-blame (21, 22). Research indicates that kids in middle and high school are most susceptible to depression (23). According to pertinent research, adolescent depression is a result of a complex interplay of genetics, social pressures, and the environment. Medical professionals should detect these potential risk factors as soon as feasible to enhance the prognosis and prevalence (24). In the future, studies combining psychiatry, psychology, and neuroscience may be conducted to gain a deeper understanding of the causes and management of adolescent depression. Though this treatment plan has not been widely employed in clinical practice, the academic community now believes that the combination of medication therapy and cognitive behavioral therapy is a good alternative for tailored treatment (22).

### 2.2 The present status of resting-state fMRI research in adolescent depression patients

In recent years, research on adolescent depression has shifted its focus to aberrant brain functional connections and their significance in pathogenesis. Several key brain areas have shown signs of dysfunction in studies. For instance, the regional homogeneity (ReHo) values are notably higher in the lingual gyrus, middle occipital gyrus, postcentral gyrus, and precentral gyrus. In contrast, ReHo values are lower in the vermis. These findings suggest that these regions might play a significant role in the onset of teenage depression (25). Research using the triple network model has shown brain network issues in adolescents with major depressive disorder (MDD). These problems mainly appear as too much connection in the salience network (SN), more connectivity between the default mode network (DMN) and SN, and weaker connections within the Central Executive Network (CEN). These network dysfunctions might cause problems with thinking and negatively impact emotional control (26). Studies of adolescents having their first depression episode with no prior treatment have found various connectivity problems in their brain networks. When resting, these adolescents showed weak links between the right amygdala, the prefrontal cortex, and the anterior cingulate cortex, which affects normal emotional control circuits (27). Also, poor connectivity between the bilateral dorsal anterior cingulate cortex and areas like the right superior frontal gyrus, frontal pole, and inferior frontal gyrus has been connected to problems in controlling emotions and a higher chance of depression (28–30). Addictive behaviors, like intense internet use, disrupt brain networks too, leading to strong emotional changes and more depression symptoms when limits are set (31). These discoveries help us understand the underlying biological changes in adolescent depression and suggest new ways to treat it.

## 3 Concepts of several effective connectivity analysis methods and their prospective applications in fMRI research

### 3.1 Dynamic causal modeling

Dynamic causal modeling (DCM) uses Bayesian algorithms to simulate the underlying neural activities from multimodal neuroimaging data. It works by employing generative and inversion methods to predict how different brain regions connect (32). The DCM model brings together neural models and hemodynamic models to forecast activity in brain areas. Using Bayesian algorithms, it compares different results to find the best one. This method helps uncover how information is combined across various brain regions under different situations. In fMRI research, the DCM model has become widely used for assessing cognitive levels of subjects and can reconstruct brain network connectivity maps based on EEG/MEG data (33, 34).

However, when dealing with huge model sets, user-defined model spaces might cause combinatorial explosion, making selection more difficult. Random effects and nonlinear modeling have helped to address these challenges, increasing flexibility and accuracy in interpreting complicated brain data (35). The efficacy of DCM is dependent on striking a balance between model biological realism and statistical inversion methods’ robustness.

Recent improvements have expanded DCM’s use in fMRI to infer effective connection across large-scale networks. Dual-state models have allowed for more exact assessment of intrinsic connection strengths, particularly in capturing neuronal dynamics (36). The relevance of self-connections in DCM analysis has frequently been neglected, emphasizing the need for additional research (37). Nonlinear equations based on the Wilson-Cowan model have also been found to outperform classic bilinear equations in DCM (38).

The Bayesian model offers unique advantages, enhancing the group-level analysis capabilities of dynamic causal modeling (DCM) and addressing inter-individual heterogeneity (39). This is particularly effective across a variety of research topics, where model analysis and predictions achieve satisfactory levels (40). Frequency domain DCM technology supports resting-state functional magnetic resonance imaging, serving as a common research paradigm and a crucial metric for assessing the effectiveness of brain network connections (41). Sparse DCM, with its high computational efficiency, allows for predictions across all brain regions, reducing the complexity associated with high-dimensional data processing (42). Stochastic DCM exhibits robustness, adeptly handling samples with high noise and data missing, making it widely used in research studies of various disease states to assess brain function (43). Cross-modal DCM helps explain how the brain interacts with external information, offering detailed insights into emotional cognition and how different senses integrate (44).

As research models get better, there have been ongoing improvements and updates in Dynamic Causal Modeling (DCM). This advancement provides new methods and tools for studying brain networks and how they process different types of information. Interest in researching DCM models is likely to stay high moving forward. Future developments will include integrating frequency domain, stochastic, and sparse modeling. These enhancements will increase the models’ ability to explain data and their practical value. This progress supports research into brain capabilities and lays the groundwork for precise treatments of neurological diseases.

**Figure 3:**
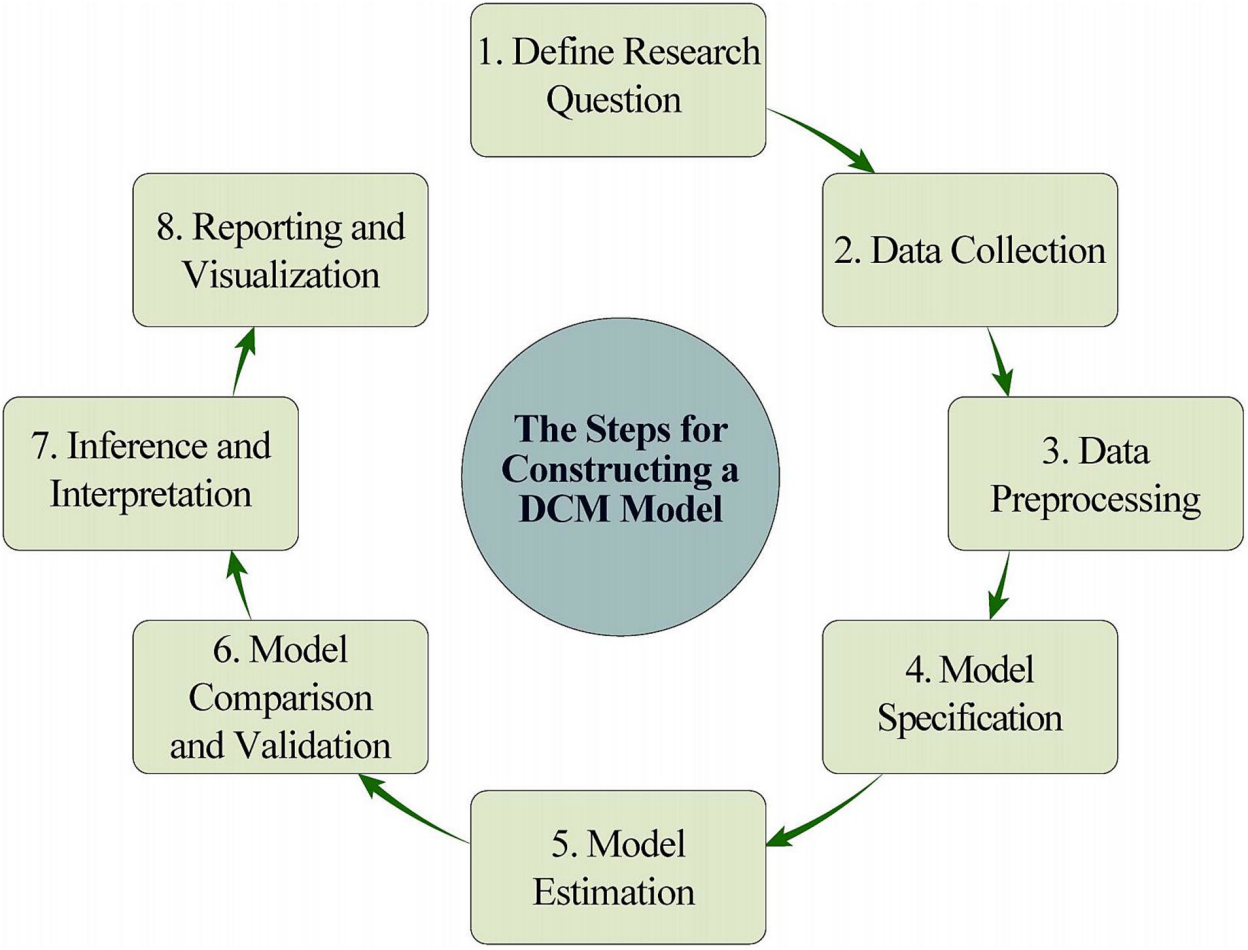
The steps for constructing a DCM model.

**Figure 4:**
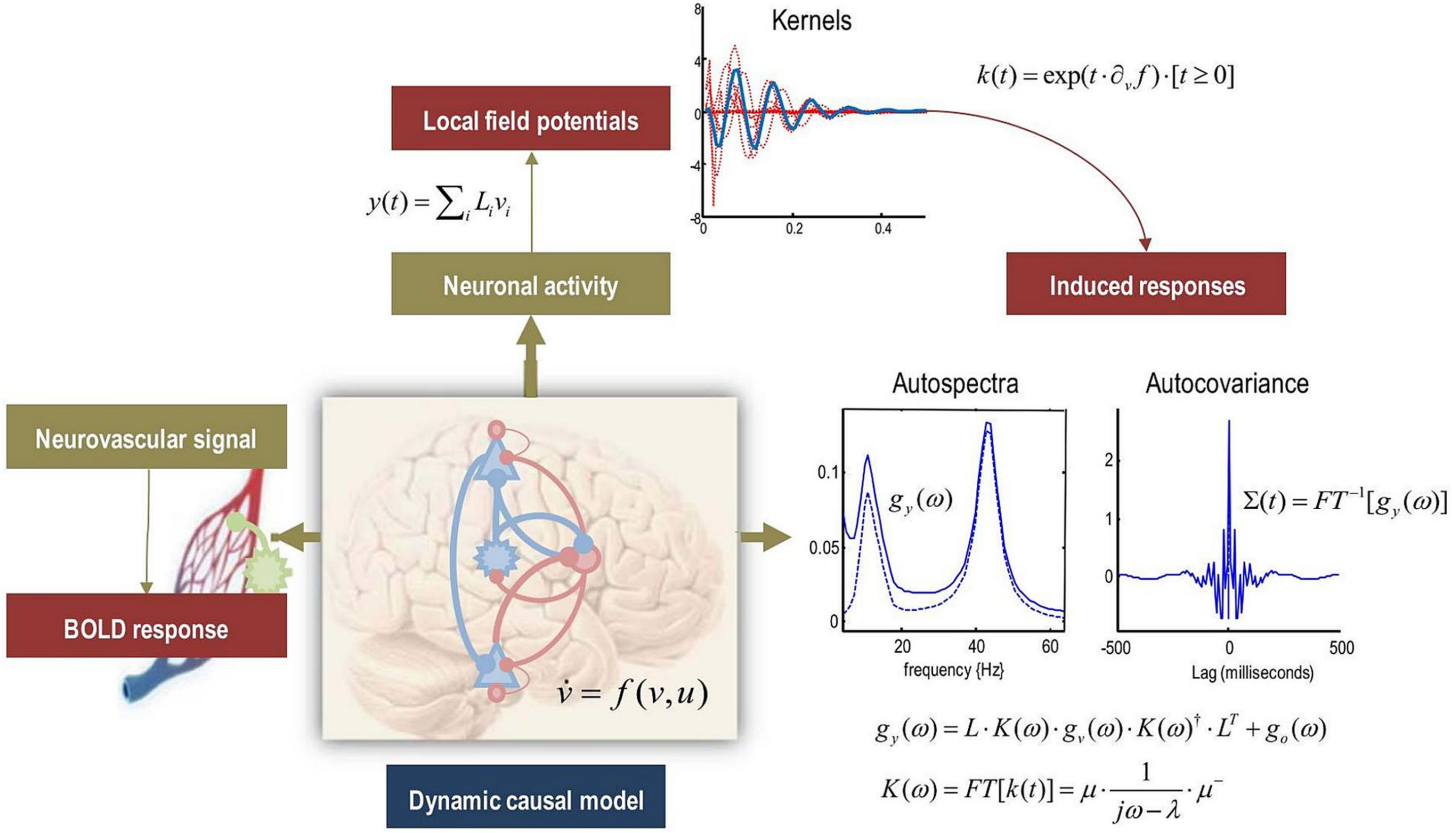
Potential of Generating Multimodal Predictions from the Same (Neuronal) Dynamic Causal Model. Adapted from Friston et al. (118), shared under the CC BY license (http://creativecommons.org/licenses/by/4.0/), DOI link: https://doi.org/10.1016/j.neuroimage.2017.02.045. The figure depicts the possibility of using the same dynamic causal model (DCM) to generate multimodal predictions. While previous figures focused on creating BOLD responses via hidden neural states, the same states can also be used to anticipate local field potentials or event-related responses, which are depicted here by a linear mapping to conventional electromagnetic fields. The top of the picture depicts a first-order kernel mapping from experimental inputs to anticipated electrophysiological responses, which represent the reaction to brief stimuli. Under the premise of local linearity, these kernels can be used to predict evoked responses caused by random variations in mean neural activity. This means that, given the spectrum density of neural fluctuations, evoked responses can be elicited. These responses are depicted on the right side of the figure using their autospectra (solid and dashed lines reflecting the presence or absence of observational noise) and corresponding autocovariance functions (the Fourier transform of the autospectra). The equations in the image describe the relationships between the first-order kernel, cross-spectral density, and covariance functions, all of which are utilized to make these predictions.

In an fMRI study based on a dynamic causal model (DCM), the research question is first identified. Then, identify the brain region of interest and its links to function. Finally, the experimental hypothesis is proposed. Subsequently, fMRI data was collected to determine the experimental paradigm and record dynamic changes in specific brain activity. The data needs to be pre-processed, including head motion correction, spatial normalization and smoothing, as well as time series data for the region of interest (ROI) to ensure the normality and validity of the results. Candidate models are built using neurophysiological assumptions, defining connections, inputs, hidden states, and outputs. Model estimate employs techniques such as Bayesian Model Averaging (BMA), with model fit determined by computing free energy. Model comparison determines the best-fitting model based on free energy and assesses parameter relevance. The analysis focuses on evaluating the causal linkages between brain areas using the best model and ensuring that they are consistent with the hypothesis. Finally, discoveries are presented through reporting and visualization, summarizing the process and laying groundwork for future research (Figures 3, 4).

### 3.2 Granger causality analysis

Granger causality analysis (GCA) is a time-series method based on multiple linear regression that is commonly used to identify effective connection between brain areas. It makes no assumptions and objectively analyses the strength and direction of brain network connections, resulting in great sensitivity and accuracy, notably in investigations of neurological and psychiatric illnesses (45). GCA’s strength stems from the use of vector autoregressive (VAR) models to forecast future values and infer causation by examining the impact of one variable on another (46). As a result, GCA has earned a reputation in neuroscience for exposing functional and directional connections. However, GCA has limits, especially when used with complicated brain systems. Its assumptions of stationarity and linearity may not always be valid, and applying it to nonlinear or nonstationary data can result in incorrect conclusions (47). The sluggish dynamics of functional magnetic resonance imaging (fMRI) signals, as well as regional hemodynamic fluctuations, call into question the assumptions concerning temporal precedence (48). GCA has its limitations, it still possesses advantages that other models cannot match, such as reducing errors in results due to incorrect hypotheses. GCA is widely used in studies on how brain mechanisms operate under cognitive deficits. To address the shortcomings of GCA, some scholars have proposed using it in conjunction with Dynamic Causal Modeling (DCM) to create complex new models. Given the many hidden layers in brain systems, some have also suggested combining DCM with Bayesian models to assess whether there is a causal relationship between hypothesis conditions and the neural system. The hidden states and coupling parameters of the DCM model reveal details about brain connectivity (49). The integration of GCA and DCM provides a foundation for deep logical analysis of brain networks, opening new avenues for research into neurological issues.

### 3.3 Structural equation modeling

The Structural Equation Modeling (SEM) has strong statistical abilities that help uncover complex relationships among various types of variables. SEM’s key advantage is its use of multiple assessment indices to evaluate and analyze latent variables with minimal measurement errors (50). It is more inclusive than traditional analytical models, enabling the exploration of multiple pathways within one model. SEM is widely used for validating theoretical models (50), making it a preferred tool in fields like psychology, sociology, and education. For instance, in psychology, SEM uses confirmatory factor analysis (CFA) to examine individual psychological changes (51); in sociology, it clarifies the relationships between different social variables (52); and in medical science, it investigates the causal links between causes of diseases and their symptoms (50). SEM’s precise and objective analysis is especially valuable in research involving complex variables and multiple hidden layers.

In recent years, Structural Equation Modeling (SEM) has been widely used in studies involving resting-state fMRI, uncovering levels of connectivity within human brain networks. Research indicates that SEM is effective in identifying causal relationships between different brain regions, facilitating precise analyses of neural network states. As a result, SEM has become a popular tool in resting-state fMRI research. It is particularly valued for assessing the connectivity of the brain’s default mode network (DMN), and for distinguishing between the brain patterns of elderly patients with depression and those of healthy groups (53). The exploratory Structural Equation Modeling (eSEM) also serves as a potent analytical tool, enhancing some functionalities of traditional SEM and offering more flexibility in modeling group data (54). Subsequently, this model framework was applied in resting-state fMRI research topics, demonstrating group connectivity pathways and predicting individual brain network connections. The expanded unified Structural Equation Model (euSEM) predicts the modulatory effects of experimental interventions on inter-regional coupling, fulfilling the analytical needs of complex brain networks (55). In summary, the prospects for SEM applications are very promising, with derivative models based on SEM enhancing understanding of brain functional network connectivity and providing data support for disease prediction and precision treatment.

### 3.4 Vector autoregressive model

The Vector Autoregression (VAR) model is commonly used for the statistical analysis of multiple variables, describing the relationships between economic variables and time series data. Its operational mechanism involves modeling variables and then combining them with their own lagged values to examine dynamic changes. With the maturation of VAR models, they now provide significant support for resting-state fMRI studies. Traditional models, which assume that connectivity among brain regions remains stable during scans, often fail to handle the real-time variations seen in imaging data. To tackle this issue, the Time-Varying VAR (TV-VAR) model was developed to allow for a flexible switch between dynamic and static monitoring (56). In functional MRI studies, choosing the right model order for VAR requires considering factors like data dimensionality, individual differences, and the goals of the experiment. While researchers commonly use first-order VAR models, higher data dimensions might require a higher model order (57). Determining the most suitable model order can be guided by criteria such as AICc and KICc, ensuring dynamic, thorough, and efficient capturing of information about brain network connectivity (58).

The analysis shows that the VAR model, along with its enhanced versions, has significantly supported resting-state fMRI research. These models excel in identifying brain networks and assessing network functional connectivity. They are adept at uncovering deep, hidden dynamic information, which opens new avenues for neurological research and lays a strong foundation for the precision treatment of neurological disorders.

### 3.5 Transfer entropy

Transfer Entropy (TE) is a key concept in information theory that mainly focuses on the direction and strength of information transfer between different time series. TE stands out from other correlation analyses because it is based on changes in probability distributions and can reveal nonlinear and lag relationships without assuming any specific dynamics of the system. This method is particularly effective for analyzing complex neural network data, whether from Electroencephalography (EEG) or functional Magnetic Resonance Imaging (fMRI). TE helps to clarify the effective connectivity between brain regions and unveils the pathways through which neural networks transmit information.

In neuroimaging, TE has been used on both static and dynamic brain networks. For example, when paired with EEG and transcranial magnetic stimulation (TMS), TE has mapped dynamic brain connection, exposing information flow during cognitive tasks (59, 60). However, TE has limitations. In nonlinear systems, it is possible to underestimate the efficiency of information transport. In multivariate networks, spurious connections can cause misinterpretations and reduce result accuracy (61). Thus, large-scale data processing using TE necessitates multivariate methodologies and stringent statistical tests in order to eliminate false positives and improve reliability.

In resting-state fMRI research, TE is commonly used as a model-free tool for assessing effective connectivity. It measures information transmission across brain areas, overcoming the assumptions made by standard models such as Granger causality and dynamic causal modeling. TE is particularly effective in detecting nonlinear interactions in brain networks (62). Studies reveal that TE is strong in noisy, linearly mixed environments, demonstrating its dependability (63). In complex brain networks, Transfer Entropy (TE) shows causal links in big, spread-out networks during cognitive tasks, helping us understand how the brain is organized (64). Extensions to TE, like phase TE and complex-valued TE, enhance its power to detect detailed interactions in certain frequency bands or multivariate systems. These are especially useful in studying brain oscillations and phase information (65, 66). While Transfer Entropy (TE) offers clear advantages over other methods, it also comes with limitations. In complex multivariate studies, the presence of spurious connections can lead to errors in the topological structure of brain networks. Additionally, TE might struggle with detecting highly nonlinear events. To maintain the objectivity of research results, it’s crucial to be thoughtful about when and how TE is applied, considering the specific conditions of each study (61).

To sum up, even though Transfer Entropy (TE) has some limits, it still serves well for studying connectivity in resting-state fMRI, especially for nonlinear connections. We can still do more research to better use TE. By combining TE with other models when we look at complex and nonlinear problems, we can learn more accurately about how brain networks work. This helps a lot in neuroscience studies.

### 3.6 Effective connectivity analysis using AI causal inference (deep learning)

In the past few years, research on AI-based causal inference has greatly improved the study of effective connectivity in neuroscience. Particularly, deep learning algorithms have started new paths for analyzing data from functional Magnetic Resonance Imaging (fMRI). Researchers have utilized these algorithms to figure out causal links between brain regions and to track brain activities. For example, they have created techniques like effective connectivity using deep reinforcement learning, known as EC-DRL (67), and systems using meta-reinforcement learning, called MetaRLEC (68). With the help of these new technologies, models of neural networks and dynamic causal learning are brought together. This combination addresses issues like high noise and small data sets. These models can also track changes over time and examine dynamic effective connectivity (dEC) (69, 70).

The use of deep learning algorithms for causal analysis has greatly advanced the study of effective connectivity, showing a lot of value in areas like brain development and neurodegenerative diseases. AI and methods driven by deep learning for causal inference are now key tools in researching effective connectivity. This marks a new era in neuroscience research and analysis. This change has made it possible to model neural dynamics more precisely and to understand complex brain functions and disorders better.

### 3.7 Directed acyclic graph

#### 3.7.1 Bayesian network

Bayesian Networks (BNs) are probabilistic graphical models that deal well with uncertainty in areas like medical diagnostics, image processing, and information retrieval (71). They start with creating a Directed Acyclic Graph (DAG) from data analysis, which helps in forming causal relationships and making Conditional Probability Tables (CPTs) for looking at data in numbers. This model works well for studying random variables in complex systems (72). Adding to the traditional BN models, researchers have developed Dynamic Bayesian Networks (DBNs) to study brain connectivity (effective connectivity) (73). Traditional BNs focus on causal structures in functional magnetic resonance imaging (fMRI) but do not include time-related connections between brain areas. DBNs, however, add a time aspect, which improves how the model handles time but can cause some loss of information. This limits its use. To overcome these issues, new forms like Gaussian DBNs and non-stationary Dynamic Bayesian Networks (nsDBNs) have been made (74, 75). DBNs are good at showing how brain connections change over time, while nsDBNs are better for dealing with time series data that changes, helping to estimate how brain regions change over different times. These improved BN models not only show how brain regions connect effectively but also help understand how the brain integrates functions and how neural dynamics change.

In conclusion, Bayesian Network models and their advancements have been crucial in neuroscience. They address the shortcomings of traditional models and offer extra data for predicting and analyzing neural connections. With the help of information technology, the potential applications of these models are expected to expand significantly.

#### 3.7.2 LiNGAM

The Linear Non-Gaussian Acyclic Model (LiNGAM) integrates linear correlation analysis with non-Gaussian noise causal analysis, featuring statistical functionality and inference capabilities (76). Faced with non-Gaussian noise data, LiNGAM can automatically generate Directed Acyclic Graphs (DAGs), delineating the causal relationships among various variables. It also distinguishes between noise and normal data, enhancing the precision of the results (77). Owing to these advantages, the model is widely applied, mapping the causal structure between different regions in brain network studies of effective connectivity. The LiNGAM model continues to evolve, capable of handling high-dimensional, nonlinear, and multi-domain data with high analytical efficiency and minimal error (78, 79). In the study of neural connectivity networks, the LiNGAM model is a viable option, though it still faces challenges in computing massive and high-dimensional data. To address this, researchers have innovatively proposed the probabilistic Linear Non-Gaussian Acyclic Model (pLiNGAM), which incorporates population data, ensuring data and causal structure stability even when fMRI data points are insufficient (80).

Undoubtedly, in the study of brain connectivity networks, LiNGAM and its optimized versions perform admirably, offering high processing efficiency and handling a large volume of data. Suitable for various types of data, these models provide stable analysis results. In the identification of brain systems, LiNGAM models effectively reveal causal relationships, making them a commonly employed tool in neuroscience research.

### 3.8 Pairwise inference methods

#### 3.8.1 Generalized partial correlation

Generalized Partial Correlation (GPC) primarily considers external factors and analyses their correlations, representing a traditional statistical method. When studying neuroimaging data, employing GPC is both feasible and scientific. Firstly, it reflects the relationships between different brain regions, and secondly, it establishes causal pathways. Building on GPC, two methods have been developed: Generalized Partial Directed Coherence (g-PDC) and Generalized Orthogonalized Partial Directed Coherence (g-OPDC) (81, 82). These methods support the study of brain connectivity during cognitive activities, reducing the risk of volume conduction artifacts and ensuring that prediction results more closely mirror actual conditions.

In summary, regarding the research topic of effective connectivity in the brain, GPC and its extensions are highly valuable. They reveal the connections and causal structures between brain regions and are adept at handling nonlinear and asymmetric data. These advantages are prominent, and the market prospects for these methodologies are promising.

#### 3.8.2 Likelihood ratio

The Likelihood Ratio (LR) evaluates the probabilities of two competing hypotheses, with one termed the null hypothesis and the other the alternative hypothesis, determining the level of support each hypothesis provides to the assessment criteria. Common evaluation methods include Activation Likelihood Estimation (ALE) and Two-stage Empirical Likelihood (TETEL), among others.

ALE combines numerous fMRI studies to generate probabilistic brain activation maps for emotional processes, making it a valuable quantitative tool in affective neuroscience (83). TETEL analyses longitudinal neuroimaging data by changing the exponentially tilted likelihood ratio to accurately estimate temporal and spatial relationships (84).

In effective connection research, LR is commonly utilized for causal inference, notably in functional and effective connectivity studies, and is a critical tool for evaluating causal linkages between brain areas (85). It enables researchers to extend beyond statistical connections and make more strong causal inferences, particularly in complex neural networks, which improves knowledge of brain function. Overall, LR improves causal inference in neuroscience, providing more precise direction and enhanced tools for future research (Table 1).

**Table 1:** Summary of effective connectivity analysis methods.

Method	Key features	Limitations	Application
DCM, SEM	Explains dynamic brain mechanisms	Limited for nonlinear or large-scale data	Dynamic modeling, network analysis
GPC, LR	Simple, suitable for nonlinear relations	Prone to spurious connections	Pairwise causal inference
Bayesian Networks, LiNGAM	Models complex causal relationships	High computational demands	Large-scale network modeling
GCA, VAR	Suitable for linear causality in time-series data	Assumes stationarity and linearity	Temporal causality analysis
TE	Captures nonlinear information flow	Sensitive to noise and spurious connections	Nonlinear network analysis
AI (e.g., EC-DRL)	Effective for noisy and complex data	Requires large datasets, hard to interpret	High-dimensional causal analysis

In this table, we provide an overview of effective connectivity analysis methods, highlighting their strengths, weaknesses, and specific use cases in studying brain networks.

### 3.9 Summary of the chapter

**Figure 5:**
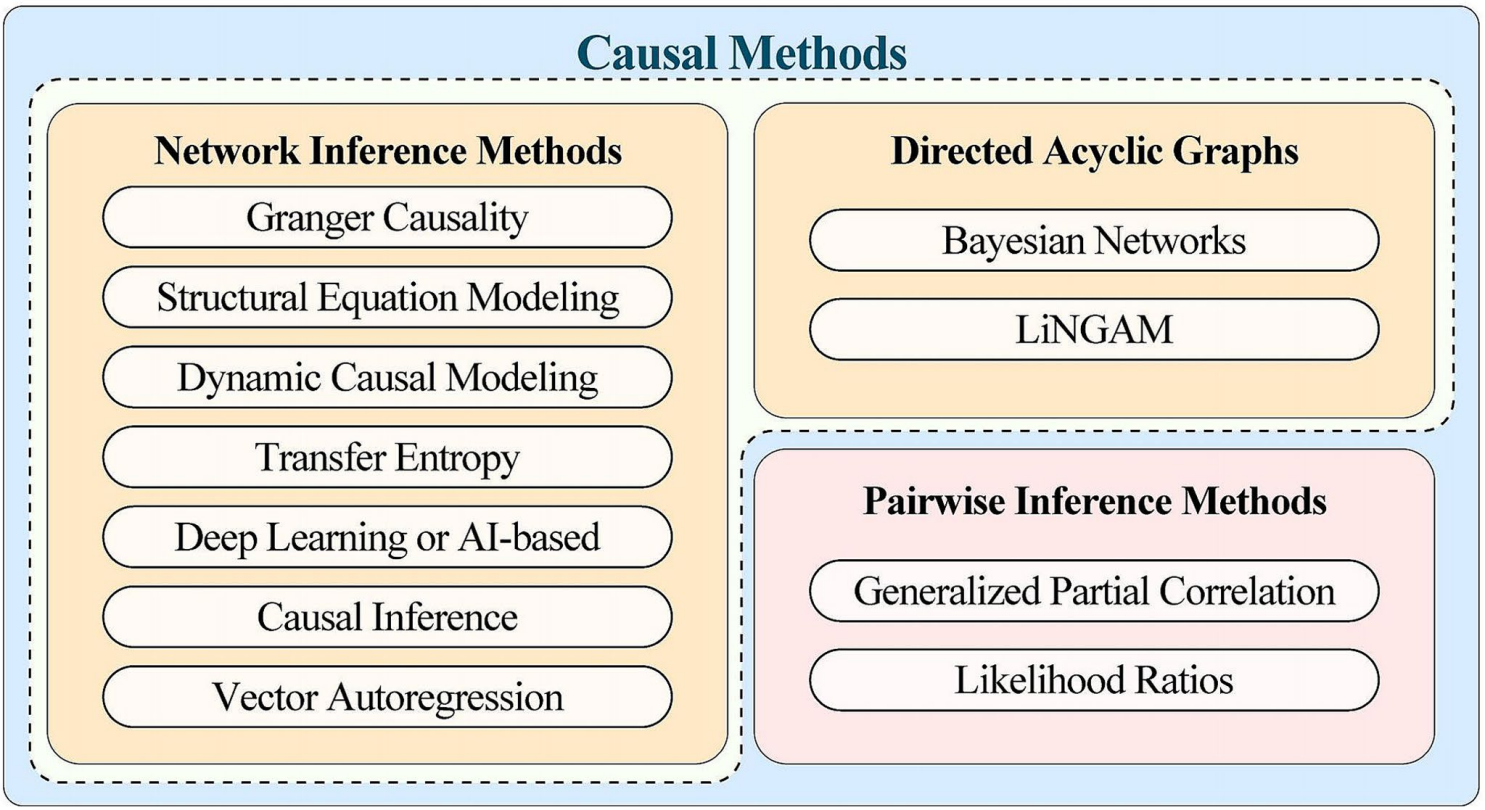
A summary of the effective join processing methods mentioned in the article. The two methods used in effective connection research are network reasoning based on a one-step multivariable process and paired reasoning based on a two-step paired process. The former deals with multiple variables, often inferring connections between multiple brain regions at once. On the other hand, pairwise reasoning based on a two-step pairwise process first deduces the connections between pairwise brain regions and then integrates them into the reasoning of the entire network.

In fMRI research, each efficient connectivity analysis method has advantages and disadvantages. Dynamic Causal Modeling (DCM) combines neuronal and hemodynamic models within a Bayesian framework, providing insights into brain function; yet, its reliance on model specification frequently results in combinatorial explosion, particularly in large-scale network research. Granger Causality Analysis (GCA) does not require a preset model, making it ideal for investigating unknown linkages; but, its assumptions of linearity and stationarity restrict its accuracy in complicated systems. Structural Equation Modeling (SEM) effectively manages measurement error but struggles with high-dimensional and nonlinear dynamic data. Vector Autoregressive (VAR) models and their time-varying extensions (TV-VAR) represent dynamic relationships in time series, but selecting the right model order for high-dimensional data presents issues. AI-based causal inference approaches, such as deep learning, perform well with high-noise and small-sample datasets, but they require big datasets and are difficult to comprehend. Directed Acyclic Graphs (DAGs), particularly Bayesian networks, are effective in modeling causal relationships in complex systems, although they are computationally intensive when dealing with huge datasets. Pairwise inference methods such as Generalized Partial Correlation (GPC) and Likelihood Ratios (LR) operate well with nonlinear and asymmetric data, but they may result in spurious connections in multivariate network analyses. In conclusion, each method has distinct applications and limits. Future research should combine several methodologies to better capture the complexity of brain networks, enhancing neuroscience and laying a solid foundation for detecting and treating neurological illnesses (Figure 5).

## 4 Abnormal effective connectivity in brain networks of depression patients

### 4.1 Abnormal effective connectivity in the default mode network

Human self-cognition is facilitated by the default mode network (DMN), a significant brain functional network that is mostly engaged during rest (86). Its central areas include the bilateral inferior parietal lobules (IPL), anterior cuneus, posterior cingulate cortex (PCC), and medial prefrontal cortex (mPFC), which are linked to social cognition, self-reflection, and emotional regulation (87). Furthermore, research has revealed that patients with significant depression have unstable DMN brain area connections, particularly between mPFC and PCC, which may result in rumination symptoms and persistently negative self-reflection (88, 89). Furthermore, in patients with recurrent depression, researchers found a more significant disruption of the effective connection (EC) between the dorsal attention network (DAN), salience network (SN), and DMN than in patients with primary depression (90). This suggests that internetwork coupling disorder is part of the pathological mechanism of depression. These results may contribute to our understanding of depression’s network connections.

### 4.2 Abnormal effective connectivity in the central executive network

The Central Executive Network (CEN), also known as the Cognitive Control Network (CCN), is a key brain functional network responsible for goal-directed behavior and complex information processing. The dorsolateral prefrontal cortex (DLPFC), which promotes goal-directed behavior and complicated information processing, is one of the key regions of the Central executive Network (CEN) (91). Along with the default mode network (DMN) and salience network (SN), CEN is a dynamic adaptive network that can be swiftly reorganized in a variety of cognitive activities and that can control the ratio of internal thought to external task demands (92). Research has demonstrated a strong correlation between cognitive decline and the loss of CEN functional connectivity, particularly in the early phases of neurodegenerative disorders and aging (93). It may be challenging for patients to suppress negative thoughts, rumination, and depressive moods since the CEN network’s self-connectivity is increased in depression patients while they are at rest, while the DMN network’s connection to the CEN network is diminished (94). Furthermore, a decrease in cognitive control is linked to disjointed connections between the medial frontal gyrus and the right superior marginal gyrus in the CEN, which may cause patients to experience difficulty making decisions and get distracted (95). In brain network switching and the processing of critical information, SN is crucial. Research has indicated that depressed individuals’ lower sensitivity to outside stimuli and difficulty switching tasks are related to the diminished effective connection between CEN and SN (96).

### 4.3 Abnormal effective connectivity in the salience network

The anterior cingulate cortex (ACC) and ventral anterior insula (vAI), along with the amygdala, hypothalamus, ventral striatum, and thalamus, form the core of the salience network (SN) (97). Integrating internal and exterior environmental perception data is a key function of the insula and anterior cingulate cortex. In order to adapt to shifting cognitive and emotional demands, SNS functionally transition between the frontoparietal network (FPN) and the default mode network (DMN) (98). Studies using functional magnetic resonance imaging (fMRI) have demonstrated a strong correlation between aberrant functional connections between brain networks and a number of mental illnesses, including major depressive disorder (MDD), schizophrenia, and schizophrenia (99). Changes in the functional and efficient connection between the Central Executive Network (CEN), DMA, SN, and the triple brain network were the specific focus of these investigations. The pathogenic process of MDD involves the weakening of the effective SN to DMN connection (EC) and the strengthening of EC from CEN to SN in patients. These changes are closely correlated with the severity of the disease (100). Furthermore, it has been discovered that the SN’s connections—particularly the EC that connects the DLPFC and the right anterior insula (rAI)—may be a significant indicator of how well a patient may respond to repeated transcranial magnetic stimulation (rTMS). Patients with strong EC between SN and CEN are more likely to respond favorably to rTMS prior to receiving therapeutic therapy (101). Our knowledge of the pathophysiology of MDD has substantially increased as a result of these investigations, and they will also aid in the development of future fMRI-based frequency-specific biomarkers.

### 4.4 Correlation between abnormally valid connections and analysis methods

In this study, we found that the abnormal connectivity characteristics of brain networks in young patients with depression are closely related to the various effective connectivity analysis methods mentioned above. The dynamic causal model (DCM) reveals a causal relationship between the weakened connection between DMN and CEN, which is consistent with established theories about the mechanisms of cognitive and emotional disorders. Granger Causality Analysis (GCA) also captures the dynamic pattern of enhanced connectivity between SN and DMN, especially its directional changes, which can give us a better understanding of network abnormalities in emotion regulation.

Structural equation models (SEM) show unique value in analyzing complex network interactions. Through SEM, we observed significant differences in the connectivity characteristics of DMN and CEN in different age groups, which suggests that the brain network of depression may have age-related evolutionary characteristics. At the same time, the analysis results of transfer entropy (TE) further enrich our understanding of the nonlinear dynamic connection between SN and CEN, especially in capturing the direction of information flow, which shows high sensitivity and applicability.

The comprehensive application of the above methods not only makes the research conclusions more comprehensive, but also provides a multi-dimensional perspective for revealing the complex mechanism behind brain network abnormalities. Combining DCM, GCA, SEM and TE techniques, we have deepened our understanding of the characteristics of brain networks in young patients with depression, and provided a reliable basis for the design and optimization of clinical interventions.

## 5 Clinical significance and future research directions

### 5.1 Findings from resting-state fMRI and EC studies for depression diagnosis

Recent advances in resting state functional magnetic resonance imaging (rs-fMRI) and effective connection (EC) have been substantial. Patients with first-episode major depressive disorder (FEDN-MDD) and those who relapsed showed significant variations in the related network ECS (102), which may indicate that depression develops gradually. The cerebellar neocortic circuit and cerebellar basal ganglia circuit in patients with major depression show significant changes, according to a study that gathered a large amount of rs-fMRI data and analyzed differences between patients with major depressive disorder (MDD) and healthy control (hc) patients. It also analyzed changes in the cerebellum and brain network (EC) in patients with major depressive disorder (103). This experiment suggests that the cerebellum brain may play a role in the onset and progression of depression; therefore, the cerebellar brain may need to be taken into account in future studies when designing experiments. Combining resting-state functional MRI (rs-fMRI) with generative models such as dynamic causal modeling (DCM) enhances the accuracy of predicting depressive episodes. The regression dynamic causal model (rDCM) is specifically designed for analyzing rs-fMRI data. It starts by evaluating whole-brain networks to provide directional estimates that are both rapid and precise, leading to recommendations for enhancing both functional and effective connectivity patterns in patients with major depressive disorder (MDD) (104). Improving research models can heighten the validity and reliability of analyses, making them more suitable for various complex scenarios. The differences discerned through EC pattern analysis (105) enable the flexible selection of model combinations in complex network studies, thereby ensuring precision and reliability in research outcomes.

These findings have revealed the cerebral origins of depression, offering crucial guidance for diagnosing and precisely treating the condition. Effective connectivity (EC) differs across various brain regions, and these differences serve as potential biomarkers for personalized and precise treatment strategies, establishing their significant clinical value. Predictive analytics and mapping of brain network relationships and structural changes have improved both diagnostic and treatment strategies, enhancing outcomes in clinical practice.

### 5.2 Issues for future research

#### 5.2.1 Sample size and heterogeneity

Current research on functional magnetic resonance imaging (fMRI) encounters several challenges, such as indeterminate study samples, uncontrollable data heterogeneity, and varied demographic standards. These issues may lead to results that lack objectivity and have replication difficulties. Due to small sample sizes, the reliability and validity of findings are often compromised, which can render the conclusions non-representative and miss important effects (106). To enhance future research, several measures could be implemented: consistently reporting effect sizes; concentrating on data variability and increasing sample sizes as necessary; and combining data from multiple sources to manage data heterogeneity effectively. For instance, Site Minimization Algorithms (SMA) could help regulate data heterogeneity (107). Another strategy might be improving data coordination procedures to ensure consistency. Expanding the diversity of fMRI study samples to include a wider range of racial, gender, and economic groups could also enhance the generalizability of the research (108). Additionally, focusing research populations more on women, adolescents, and diverse racial groups could broaden the scope and impact of the findings.

In summary, future research using rs-fMRI and EC should emphasize the quantity, diversity, and heterogeneity of samples. This approach will help ensure the objectivity, generalizability, and reliability of the findings.

Furthermore, the potential preventive effects of counseling and psychological therapy were not evaluated in this study. Despite the fact that these therapies are frequently used in practice to stop or lessen symptoms during episodes, we were unable to thoroughly assess their effects in this study because of a lack of pertinent data. In order to further examine psychological therapy and counseling interventions’ possible significance in symptom management, future research should think about incorporating them into the evaluation.

#### 5.2.2 Interdisciplinary collaboration and technology integration

Research in a number of fields, including clinical medicine, computer science, neurology, and data science, may benefit from the application of rs-fMRI technology. As a result, in order to handle the complexity and data processing issues, the study team needs the necessary equipment (109). Future studies should require relevant researchers to become proficient in a variety of technologies. Creating uniform methods for preprocessing and tools for analysis is crucial as we deal with more data and complex signals (110). This requires new algorithms and also teamwork across different fields in collecting, processing, and analyzing data. Techniques like dynamic rs-fMRI and multiband imaging offer new insights into the time-related and spatial changes in brain networks. Their practical application is dependent on interdisciplinary efforts in noise reduction, parameter optimization, and sophisticated methods such as graph theory-based network analysis, which have shown promise in finding complex brain structures (111). However, future development of these technologies necessitates greater interdisciplinary collaboration, particularly in assessing biological relevance. As rs-fMRI’s potential for identifying and treating psychiatric and neurodegenerative illnesses rises, interdisciplinary collaboration becomes increasingly important (112). Despite better understanding of cognitive brain networks, using this knowledge faces challenges like signal differences and not enough standardized studies. Machine learning and big data analytics might help solve these problems (113). However, working closely across different fields is essential to make sure these technologies work well and are used widely. Research findings point out that understanding and treating depression are closely connected to the study of EC. Future investigations might slowly focus more on brain paths that are part of depression. This could make research outcomes more useful in real-world settings, helping to offer more support in clinics. Technological progress, like using machine learning to mimic the EC of brain networks, allows for predicting EC levels and checking how patients react to different treatments like drugs, therapy, and brain stimulation methods. Studying EC helps medical experts precisely find and tailor treatment strategies that suit the forecasted outcomes for people with depression. This is very important for young people with depression, as they often deal with a lot of school-related stress. By examining the EC in their brain areas and using different imaging types together, doctors can assess brain function, show pictures of brain structure issues, and thus create more accurate treatment plans.

Research on effective connectivity supports the medical treatment of depression by bringing together different research methods and encouraging collaboration across various fields. For example, merging machine learning algorithms with neuroimaging helps create precise treatment plans for each person. It is only by working together that technologies like resting-state fMRI and effective connectivity can keep improving. This will lead to better personalized medical services.

### 5.3 Comparisons of effective connectivity findings across age groups

Though comparisons with brain network features in other age groups, particularly older patients, can offer more information, this study concentrated on effective connectivity (EC) deficits in adolescents with depression. According to studies, teenage patients’ aberrant brain networks are primarily dynamic alterations, with a particularly strong link between DMN and SN. The instability of emotional and cognitive control during the adolescent brain’s maturation period may be connected to this phenomena (114). On the other hand, the internal function of the DMN, particularly the connection between the mPFC and the PCC, was significantly diminished in older patients with anomalies in their brain networks. The substantial impacts of aging on brain network function are reflected in this long-term degeneration, which is frequently accompanied by cognitive decline and increased rumination (115).

Even though the two patient groups have different abnormalities, DMN and SN dysfunctions are significant in both adolescents and older patients, indicating that depression may have some fundamental brain mechanisms in common. While the reduced connections in older patients are more likely to be due to long-term pathogenic processes, the improved dynamic connections in teenagers may be a reflection of the plasticity of brain networks. This mix of distinctions and similarities offers hints for investigating the brain processes underlying depression in various age groups.

To ascertain whether adolescent brain network abnormalities are primarily caused by adolescent brain network remodeling or are influenced by outside psychological pressure, future research should investigate the mechanism behind these abnormalities in greater detail. The development trajectory of depression, the scientific foundation for the creation of stratified intervention strategies, and the creation of individualized treatment plans based on the brain network characteristics of patients of various ages are also anticipated to be revealed by comparing the brain network characteristics of elderly and adolescent patients.

### 5.4 Chapter summary

This section talks about how rs-fMRI and EC analysis are important in diagnosing and treating depression. It is evident that despite being current research challenges, these areas are pivotal for future investigations. They play a crucial role in identifying neurobiological markers of depression, particularly in predicting anomalies within brain functional networks. Current research has limitations, such as insufficient sample sizes, significant data variability, inadequate models, and restricted interdisciplinary research, indicating substantial room for improvement in future studies. Specifically, the volume of research on effective connectivity in adolescent depression is limited, with few references and inadequate sample sizes, affecting the reliability and validity of conclusions. Additionally, data variability, such as differences in depression severity and brain connectivity models between genders, impacts the objectivity of results (116). Currently, commonly used models in the field include dynamic causal modeling (DCM) and Granger causality analysis (GCA). These linear models struggle with analyzing dynamic and complex neural data, such as nonlinear or dynamically interactive data. Improper preprocessing can also increase result repeatability and decrease conclusion validity. The time resolution of rs-fMRI is limited, making it difficult to capture rapid dynamic activities. To address these issues, increasing sample sizes, improving data heterogeneity, and utilizing multimodal imaging analysis to obtain comprehensive brain activity maps are recommended, thereby providing more robust data support for precise diagnosis and treatment of depression.

## 6 Conclusion

Studies on resting-state functional MRI (rs-fMRI) and effective connectivity (EC) have built a solid base, both practical and theoretical, for understanding depression. Research has linked the start of depression to problems in EC within brain networks, especially in the Default Mode Network (DMN), Central Executive Network (CEN), and Salience Network (SN). Looking at the connections in these areas helps reveal the complex biology of depression and may provide markers to better diagnose and treat it. Current studies show that lower connectivity in the DMN, CEN, and SN can mess up how emotions are controlled and increase thinking problems. These issues can lead to ongoing negative feelings and deep thinking about the same sad thoughts. Also, problems in the SN can disturb how emotions are regulated, making depression more likely. These insights help shape technical strategies for treating depression (117).

This paper investigates how three main brain networks connect: the Default Mode Network (DMN), Central Executive Network (CEN), and Salience Network (SN). It looks at young people with depression. By comparing, it shows that understanding these connections can help in diagnosing and treating this condition. At first, different models for studying connections were combined with resting-state fMRI. This was to find unusual connections that might be early signs of trouble. The research finds these connections are very important for accurate treatments. They could help stop depression in young people before it starts.

Future studies need to look at a couple of important points. First, we need more people in the studies, and we must control the differences in the data carefully. A small number of participants makes it hard to apply the findings widely, and too much difference in the data can make the results less valid and reliable. Second, it is becoming very important to use ideas from different fields and new technologies. By using many types of imaging together, we can move beyond just one way of studying things. This will help us understand brain activity better and support making treatment plans that are accurate and specific to each person.

Finally, rs-fMRI and EC analysis have revealed the brain mechanisms behind depression. This is both a main area of interest and a challenge in research today. Going forward, we need to tackle problems like not having enough participants, varied data, studies that only use one scientific approach, and old research methods. A deeper understanding of brain network functions and connectivity will contribute significantly to the precision medicine of neurological disorders.

## Data Availability

The original contributions presented in the study are included in the article/supplementary material, further inquiries can be directed to the corresponding authors.
